# Pre-miR-27a rs895819A/G Polymorphisms in Cancer: A Meta-Analysis

**DOI:** 10.1371/journal.pone.0065208

**Published:** 2013-06-07

**Authors:** Qian Xu, Cai-yun He, Jing-wei Liu, Yuan Yuan

**Affiliations:** Tumor Etiology and Screening Department of Cancer Institute and General Surgery, the First Affiliated Hospital of China Medical University, the Key Laboratory of Cancer Etiology and Prevention in Liaoning Province, Shenyang, Liaoning Province, China; Ohio State University Medical Center, United States of America

## Abstract

**Background:**

MicroRNAs (miRNAs) negatively regulate the 3′ untranslated region (3′-UTR) of coding genes by suppressing translation or degrading mRNAs, and they act as oncogenes or tumor suppressors. Recently, several studies investigated the association between pre-miR-27a rs895819 polymorphism and the risks of various cancers, but the results were inconsistent.

**Methodology/Principal Findings:**

We conducted a meta-analysis of 13 studies that included 6501 cancer cases and 7571 controls to address this association. Overall, this meta-analysis showed that the pre-miR-27a rs895819 A/G polymorphism was not statistically associated with cancers risk in all genetic models. In the stratified analysis by cancer types, when compared with the ancestral A allele, individuals with the variant G allele was consistently associated with reduced risks of breast cancer (OR = 0.92, 95% CI = 0.85–0.99), renal cell cancer (OR = 0.81, 95% CI = 0.67–0.97) and nasopharyngeal cancer (OR = 0.84, 95% CI = 0.72–0.97). Inversely, individuals with the heterozygote AG was associated with an increased risk of digestive tract cancers compared with AA genotype (AG vs. AA: OR = 1.16, 95% CI = 1.01–1.32). In the stratified analysis by ethnicity, the pre-miR-27a rs895819 polymorphism showed statistically significant association with decreased risks of cancers in Caucasians (G vs. A allele: OR = 0.90, 95% CI = 0.83–0.97; AG vs. AA: OR = 0.84, 95% CI = 0.75–0.94; AG/GG vs. AA: OR = 0.85, 95% CI = 0.76–0.94) but not in Asians.

**Conclusion/Significance:**

This meta-analysis suggests that the pre-miR-27a rs895819 polymorphism may contribute to the susceptibilities of some specific-type of cancers, including breast cancer, renal cell cancer, nasopharyngeal cancer and digestive tract cancers, as well as the susceptibilities of cancers in Caucasians to some extent.

## Introduction

MicroRNAs (miRNAs) are RNAs that are 18–23 nucleotides long that participate in the transcriptional regulation of eukaryotic genes [1], which is associated with post-transcriptional modifications that lead to the mRNA’s degradation or the translational repression of the targeted genes [2]. miRNA genes are transcribed to a hairpin-shaped RNA form named pri-miRNA [3], which is processed by an endonuclease, Drosha, to form a pre-miRNA that is 60–70 nucleotides long [4]. The pre-miRNAs are cleaved by an endonuclease, Dicer, to form the mature miRNA, which functions as a gene regulator [5]. The variation of the pri-, pre-, and mature miRNA through physiological processes may cause the incidence and development of a tumor.

miRNAs are transcribed from a gene intron and/or intergenic region. Polymorphisms exist in the pri-, pre-, and mature miRNA genes that can potentially influence the processing of maturing miRNAs, broadly affecting the miRNA function [6]. The first research focused on pre-miRNA polymorphisms screened 323 single nucleotide polymorphisms (SNPs) in 227 miRNA genes according to bioinformation by Duan et al. [6]. From then on, scholars all focused on 3 SNP sites in the field of the association between miRNA polymorphism and cancers, hsa-pre-miR-146a rs2910164, hsa-pre-miR-196a2 rs11614913, and hsa-miR-499 rs374644, and several meta analyses reported that these 3 sites were associated with cancer risks [7]–[16]. The miR-27a rs895819 A/G polymorphism is located in the loop of the pre-miRNA, and the variation from A to G could cause a change in the minimum free energy (MFE). Therefore, this SNP may affect the function of the miRNA to some extent. However, the results regarding its association with cancer risk are conflicting. The majority of studies reported that the variant allele could decrease the cancer risk [17]–[19], but some studies reported different viewpoints. A few studies reported that the association between the variant G allele and cancer risk was insignificant; these studies denied the protective role of the G allele [20]–[22]. Moreover, some studies reported that the variant G allele was a risk allele. For example, 2 studies using samples from the same continent analyzed the AG and GG genotypes for the risk of gastric cancer and compared the risk with that for the AA wild-type genotype. One study found that the AG and GG genotypes increased the risk of gastric cancer [23], and the other study found the opposite result [24]. Therefore, the comprehensive analysis that integrates all individual studies is still required. To enhance the efficiency of meta-analysis on the risk of cancers and investigate whether this rs895819 polymorphism was associated with the gastric cancer risk, an unpublished case-control study on gastric cancer that was performed by Qian Xu et al at the Tumor Etiology and Screening Department of Cancer Institute and General Surgery in the First Affiliated Hospital of China Medical University, Shenyang, China was collected. Overall, 13 datasets consisted of 6501 cases with cancers and 7571 controls were included in our study. These data should be combined to expand our understanding of the pre-miR-27a rs895819 SNP, which may provide some evidence for the future research. Thus, in this study we conducted a meta-analysis to combine all of the available studies and validate whether the miR-27a A/G polymorphism contributes to the risks of cancers.

## Methods

### Publication Search

A systematic literature search was performed for all articles on the association between pre-miR-27a rs895819 polymorphism and cancer risks up to March 15th, 2013. The PubMed databases, Chinese National Knowledge Infrastructure (CNKI) and Web of Science were used simultaneously. The following key words were used: “miR-27a”, “cancer/carcinoma/tumor/neoplasm”, “rs895819” and “polymorphism” by two independent investigators Qian Xu and Cai-yun He. All the studies met the following criteria: (1) case-control study; (2) the association between miR-27a polymorphism and cancer risks was explored; and (3) genotype frequency was available. The major exclusion criteria were: (1) duplicate data, (2) the study only for cancer samples or for precancerous disease compared with controls, and (3) the subjects in control group were high-risk individuals with some gene mutations.

### Data Extraction

Two of the authors (Qian Xu and Cai-yun He) extracted all data independently, complied with the selection criteria, and reached a consensus on all items. The following items were collected: first author’s name, year of publication, country of origin, ethnicity, cancer type, language, genotyping method, source of control groups (population- or hospital-based), and total number of cases and controls, and genotype distributions in cases and controls. Meanwhile, we categorized gastric cancer, colorectal cancer and liver cancer into ‘digestive tract cancer’ for the stratified analysis.

### Statistics

Firstly, the genotype frequencies of miR-27a polymorphism for Hardy-Weinberg equilibrium (HWE) were assessed by the Chi-square test in controls and a *P*<0.05 was considered as significant disequilibrium. The strength of the association between the miR-27a polymorphism and cancer risks was estimated by odds ratios (ORs) with 95% confidence intervals (CIs). Statistical heterogeneity among studies was tested by Chi square-based Q test and I^2^ [25]. Heterogeneity was considered significantly when *P*<0.10 was observed in Q test, and I^2^ was used to qualify variation in OR attributable to heterogeneity. When heterogeneity exists, a random-effect model based on DerSimonian and Laird method was used to calculated the pooled OR of each study [26]; otherwise, a fixed-effect model based on the Mantel-Haenszel method was employed [27]. Generally, we first evaluated the risks between G allele and cancer risks compared with that for A allele in the codominant model (AG versus AA, GG versus AA), dominant model (AG/GG versus AA) and recessive model (GG versus AG/AA), and allelic comparison (G versus A). We also performed stratification analyses on cancer type (divided into digestive tract cancer, breast cancer, nasopharyngeal cancer and renal cell cancer) and ethnicity (Asian and Caucasian). Meta-regression was further performed to detect the source of heterogeneity. The between-studies variance (τ^2^) was used to quantify the degree of heterogeneity between studies and the percentage of τ^2^ was used to describe the extent of heterogeneity explained [28]. The Begg’s rank correlation method was used to statistically assess publication bias [29] (*P*<0.10 was considered as statistically significant). All analyses were done using STATA software, version 11.0 (STATA Corp., College Station, TX, USA), and all tests were two-sided.

## Results

### Characteristics of the Studies

**Figure 1:**
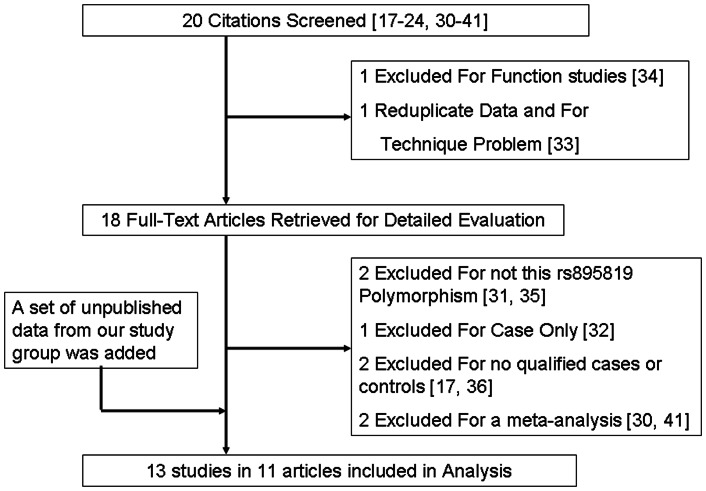
Studies identified in this meta-analysis based on the criteria for inclusion and exclusion.

A total of 20 articles were found by literature search from the PubMed, CNKI and Web of Science [17]–[24], [30]–[41], using different combinations of key terms. As shown in Figure 1, we excluded nine studies (one was function study [34], one was reduplicate data and showed technique problem [33], two were not studied for the rs895819 polymorphism [31], [35], one is case-only study [32], two had no qualified cases or controls [17], [36], and two were meta-analyses about the rs895819 SNP [30], [41]). In addition, our research group provided this meta-analysis with a set of unpublished data regarding the association between rs895819 polymorphism and gastric cancer risk. Accordingly, a total of 13 case-control studies extracted from 11 articles and a set of our unpublished data that met our inclusion criteria were included in the final meta-analysis [18]–[24], [37]–[40], which consisted of 6501 cancer cases and 7571 cancer-free controls. The characteristic of each included study was presented in Table 1. Of the enrolled 13 studies, all were matched for age, sex except 4 studies for breast cancer that did not need to match sex; 10 studies investigated in Asians and the other 3 studies investigated in Caucasians; controls in 7 studies were hospital-based and controls of the other studies were population-based or mixed subjects; genotyping methods included PCR-RFLP (3 studies), TaqMan (3 studies), PCR-based single base extension (5 studies) and PCR-direct sequencing (2 studies). 11 out of 13 studies checked genotypes for quality control. Genotype distribution of controls in all studies was consistent with Hardy-Weinberg equilibrium.

**Table 1:** Characteristics of literatures included in this meta-analysis for miR-27a rs895819A/G polymorphism.

First author	Year	Country	Ethnicity	Cancer type	Language	Genotyping method	Source of control groups	Sample size		Case				Control				*P* of HWE
								Case	Control	AA	AG	GG		AA		AG	GG	
Qian Xu	2013*	China	Asian	Gastric cancer	Chinese*	Sequencing	PB and HB mixed	222	305	115	100	7			173	110	22	0.437
Danni Shi	2012	China	Asian	Renal Cell Cancer	English	TaqMan	HB	594	600	334	213	47			288	262	50	0.373
Renata Hezova	2012	Czech	Caucasian	Colorectal cancer	English	TaqMan	HB	197	212	88	86	23			93	94	25	0.867
Irene Catucci	2012	Italy	Caucasian	Breast cancer	English	TaqMan	PB	1025	1593	547	388	90			803	633	157	0.051
Yuan Zhou	2012	China	Asian	Gastric cancer	English	MassARRAY	HB	295	413	166	122	7			214	167	32	0.941
Mingwu Zhang	2012	China	Asian	Breast cancer	English	PCR-RFLP	PB	245	243	60	144	41			75	109	59	0.123
Mingwu Zhang	2012	China	Asian	Colorectal cancer	Chinese	PCR-RFLP	PB	463	468	248	178	37			270	166	32	0.351
Peirao Li	2011	China	Asian	Liver cancer	Chinese	SNPstream	HB	401	459	190	178	33			233	190	36	0.751
Peirao Li	2011	China	Asian	Nasopharyngeal cancer	Chinese	SNPstream	HB	801	1022	443	300	58			505	431	86	0.658
Ping Zhang	2011	China	Asian	Breast cancer	Chinese	MassARRAY	Not mentioned	376	190	196	150	30			106	70	14	0.605
Wenzhang Wang	2011	China	Asian	Liver cancer	Chinese	MassARRAY	HB	361	340	186	175†				162	178†		0.407
Qingmin Sun	2010	China	Asian	Gastric cancer	English	PCR-RFLP	HB	304	304	115	135	54			145	119	40	0.053
Rongxi Yang	2010	Germany	Caucasian	Breast cancer	English	Sequencing	PB	1217	1422	576	486	127			605	660	151	0.142

HB, hospital based; PB, population based; HWE, Hardy-Weinberg equilibrium; PCR-RFLP, polymerase chain reaction-restriction fragment length polymorphism.

* the study year when this unpublished data was finished in a Chinese population.

† only the data of dominant model could get, so this study was only analyzed when the dominant model was used.

### Quantitative Synthesis

First, all the eligible studies were pooled to estimate the association strength of mir-27a rs895819 polymorphism with the overall risk of all types of cancers. No statistically significant association was found in all genetic models (Table 2). We further performed stratification analysis based on different cancer types and different ethnicities (Table 2).

**Table 2:** Pooled ORs and 95% CIs of the overall and stratified meta-analyses.

Variables	N	AG vs. AA			GG vs. AA			AG/GG vs. AA				GG vs. AA/AG			G vs. A		
		OR(95% CI)	*P* a	I^2^(%)	OR(95% CI)	*P* a	I^2^(%)	N^d^	OR(95% CI)	*P* a	I^2^(%)	OR(95% CI)	*P* a	I^2^(%)	OR(95% CI)	*P* a	I^2^(%)
All cancers	12	1.01	0.894	70.0 c	0.90	0.080	46.1^b^	13	0.98	0.685	65.2 c	0.91	0.123	43.8^b^	0.96	0.403	58.8 c
		(0.88–1.16)			(0.79–1.01)				(0.86–1.10)			(0.81–1.03)			(0.88–1.05)		
Cancer type																	
Digestive tractcancer	6	**1.16**	**0.031**	0.0 b	0.90	0.667	70.7 c	7	1.08	0.181	43.0 b	0.86	0.471	67.6 c	1.06	0.403	58.4 c
		**(1.01–1.32)**			(0.57–1.43)				(0.96–1.21)			(0.56–1.31)			(0.91–1.23)		
Breast cancer	4	1.01	0.945	77.1 c	0.88	0.154	0.0 b	4	0.96	0.706	66.2 c	0.90	0.210	16.4 b	**0.92**	**0.023**	0.0 b
		(0.78–1.30)			(0.74–1.05)				(0.79–1.18)			(0.76–1.06)			**(0.85–0.99)**		
Renal Cell Cancer	1	**0.70**	**0.004**	/	0.81	0.336	/	1	**0.72**	**0.004**	/	0.95	0.790	/	**0.81**	**0.019**	/
		**(0.55–0.89)**			(0.53–1.24)				**(0.57–0.90)**			(0.62–1.43)			**(0.67–0.97)**		
Nasopharyngealcancer	1	**0.79**	**0.020**	/	0.77	0.148	/	1	**0.79**	**0.012**	/	0.85	0.357	/	**0.84**	**0.018**	/
		**(0.65–0.96)**			(0.54–1.01)				**(0.66–0.95)**			(0.60–1.20)			**(0.72–0.97)**		
Ethnicity																	
Asian	9	1.08	0.410	72.6 c	0.90	0.480	60.0 c	10	1.03	0.761	69.3 c	0.86	0.261	57.6 c	0.99	0.895	67.2 c
		(0.90–1.31)			(0.68–1.20)				(0.87–1.21)			(0.66–1.12)			(0.87–1.13)		
Caucasian	3	**0.84**	**0.003**	3.7 b	0.87	0.143	0.0 b	3	**0.85**	**0.002**	0.0 b	0.95	0.549	0.0 b	**0.90**	**0.009**	0.0 b
		**(0.75–0.94)**			(0.73–1.05)			**(0.76–0.94)**				(0.80–1.13)			**(0.83–0.97)**		

OR: odds ratio; CI: confidence interval. The results were in bold, if the 95% CI excluded 1 or *P*<0.05.

a the statistical significance of the pooled OR was determined by the Z test;

b No statistical significance was found by the heterogeneity test, then the fixed-effects model was adopted here.

c confirmatory analyses with a random-effect model was used if there was significant heterogeneity.

d when the dominant model was analyzed, the data studied by Wenzhang Wang (2011) was added.

In the stratification analysis of cancer type, the variant G allele was observed to be statistically associated with a decreased risk of breast cancer (OR = 0.92, 95% CI = 0.85–0.99). Since only one study was conducted in renal cell cancer or nasopharyngeal cancer [19] [38], the pooled OR could not be appraised on these two cancer types. Based on single study, we observed that the AG heterozygote and AG/GG genotypes were consistently associated with decreased risks of renal cell cancer (AG vs. AA:OR = 0.70, 95% CI = 0.55–0.89; AG/GG vs. AA: OR = 0.72, 95% CI = 0.57–0.90) and nasopharyngeal cancer (AG vs. AA:OR = 0.79, 95% CI = 0.65–0.96; AG/GG vs. AA: OR = 0.79, 95% CI = 0.66–0.95) when compared with the wild-type AA genotype. In addition, the G allele was statistically associated with decreased risks of renal cell cancer (OR = 0.81, 95% CI = 0.67–0.97) and nasopharyngeal cancer (OR = 0.84, 95% CI = 0.72–0.97) compared with the A allele. These observations insinuated a reverse association of G allele with the risks of those tumor types. Nevertheless, the comparison of AG versus AA in digestive tract cancer subgroup showed an opposite result. The AG genotype was associated with an increased risk of digestive tract cancer (AG vs. AA genotype: OR = 1.16, 95% CI = 1.01–1.32).

In the stratification analysis of ethnicity, when compared with the ancestral AA genotype, the AG heterozygote and AG/GG genotypes were associated with a decreased risk of cancers in Caucasians subgroup (AG vs. AA: OR = 0.84, 95% CI = 0.75–0.94; AG/GG vs. AA: OR = 0.85, 95% CI = 0.76–0.94) but not in Asians subgroup. We observed the same association in the allelic comparison (G allele vs. A allele: OR = 0.90, 95% CI = 0.83–0.97).

### Heterogeneity

The heterogeneity within each subgroup was shown in Table 2. A part of comparisons showed slight or moderate heterogeneities between studies. We subsequently conducted sensitivity analyses to explore individual study’s influence on the pooled results by the removing one study at a time from pooled analysis (Table S1). The results showed no individual study affected the pooled OR significantly. We therefore further explored the source of heterogeneity by cancer type, ethnicity, language, and genotyping method with meta-regression in allelic comparison (G vs A). Meta-regression results revealed that cancer type (*P* = 0.023) but not ethnicity (*P* = 0.452), language (*P* = 0.347), or genotyping method (*P = *0.100) contributed to the source of heterogeneity. Additionally, cancer type could explained 85.29% of the between studies variance (τ^2^).

### Publication Bias

Begg’s rank correlation was conducted to evaluate publication bias. In the comparison of AG vs. AA and dominant model (AG/GG versus AA), a slight publication bias was observed (AG vs. AA: *P* = 0.020; AG/GG versus AA: *P* = 0.088, respectively, Table 3), insinuating potential publication bias due to a language bias, inflated estimates by a flawed methodological design in smaller studies, and/or a lack of publication of small trials with opposite results.

**Table 3:** The results of Begg’ s test for the publication bias.

Comparison type for miR-27a rs895819	Begg’ s test	
	Z value	*P* value
AG vs. AA	2.33	0.020
GG vs. AA	−0.41	0.681
AG/GG vs. AA	1.71	0.088
GG vs. AA/AG	−1.10	0.273
G vs. A	0.69	0.493

## Discussion

miR-27a, which is located in an intergenic region of chromosome 19 (chr19∶13947254–13947331), had up-regulated expression in many tumors [42] and was considered to be an oncomir [43]–[45]. Using the single-stranded conformational polymorphism-polymerase chain reaction (PCR-SSCP) method Tomiyasu Arisawa discovered in 2007 that there was a polymorphism in the flanking region of miR-27a gene, and the variant G allele frequency reached 34.6% [36]. In the same year, Ranhui Duan et al. employed a bioinformatic method to screen 323 SNPs, and emphasized the importance of this rs895819 SNP because of its location in the pre-miRNA sequence [6]. Since then, several studies of this SNP emerged in succession. In 2010, Tair Kontorovich et al., using a Sequenom MassARRAY, screened 42 miRNA-related SNPs and found that AG heterozygote of miR-27a rs895819 had a lower risk for breast/ovarian cancer among Jewish women [17]. At almost the same time, Rongxi Yang et al. used a sequencing method to observe that the variant G allele significantly decreased familial breast cancer risk in a German population [18]. Following that, it was suggested that the AG and GG genotypes had a decreased renal cell cancer risk in a Chinese population compared with the AA wild-type genotype [19]. However, some studies hold different viewpoints; a few studies considered the relationship between the variant G allele and the risk of tumor to be insignificant [20]–[22]. Moreover, some researchers thought that the variant G allele was a risk allele. Two inconsistent studies investigated the risk of gastric cancer using samples from the same country. Qingmin Sun et al. found that the AG and GG genotypes were associated with an increased risk of gastric cancer and an increased expression level of miR-27a [23], but Yuan Zhou observed the opposite result [24].

According to our meta-analysis, A→G variation at miR-27a rs895819 polymorphism site did not exert significant genetic effect on cancer risk in the overall analysis (Table 2). We further performed stratification analysis based on cancer type and ethnicity. In breast cancer subgroup analysis integrating 4 studies, subjects carrying G allele of rs895819 demonstrated reduced cancer risk. Additionally, one study about renal cell cancer [19] and one study on nasopharyngeal cancer [38] could not be incorporated into other cancer types and thus were analyzed separately as single cancer type in this meta-analysis. For renal cell cancer and nasopharyngeal cancer, association analyses under codominant model (AG versus AA), dominant model (AG/GG versus AA) and allelic comparison (G vs. A) consistently supported that this polymorphism was related to decreased risks of both cancers. The slight difference of ORs and 95% CI between our meta-analysis and their original studies was probably due to unavailable raw data for adjustment of other factors. All these findings indicated that this polymorphism may be associated with decreased risks of specific cancers including breast cancer, renal cell carcinoma and nasopharyngeal carcinoma.

In subgroup analysis of digestive tract cancer, however, we observed that AG genotype was associated with increased cancer risk. As for the single original study, Sun et al ’s [23] reported a statistically positive association of rs895819 polymorphism with gastric cancer risk; four studies [23], [37], [38] (Qian Xu, 2013^*^) showed that this polymorphism tended to be positively associated with higher risks of digestive tract cancers, although they did not reach statistical significance. In this meta-analysis, we incorporated and evaluated the available data of this polymorphism with digestive tract cancer risk, which may demonstrate a relatively stable result. The difference of the association of this polymorphism with various cancers may result from distinct cell origin and pathogenetic mechanisms of different cancers.

To better elucidate the association of this polymorphism with digestive tract cancer, we added a set of unpublished data which was a case-control study including 222 gastric cancer cases and 305 superficial gastritis patients as controls in Chinese. The control group contained participants from a health check program for gastric cancer screening in Zhuanghe region of Liaoning Province, China and patients who underwent gastroscopic examination in the First Affiliated Hospital of China Medical University, Shenyang, Liaoning Province, China. All the 527 subjects were diagnosed independently by two professorial pathologists. By integrating currently-published 6 studies and our newly-unpublished data, we found for the first time that this polymorphism may be associated with digestive tract cancer, which is also one of the differences between our meta-analysis and previously published meta-analyses. Furthermore, considering the relatively obvious heterogeneity observed in this meta-analysis even after employing sensitivity analyses and subgroup analyses, we carried out meta-regression to further explore the source of heterogeneity. Interestingly, the results of meta-regression indicated the heterogeneity mainly resulted from different cancer types and this variance could explain the majority of heterogeneity origin. Therefore, the different effects of this polymorphism on various cancers should not be overlooked and its specific effects on different cancer types are warranted to be clarified in future research.

Limitations of this study should also be noted. First, we pooled the data using unadjusted information, whereas a more precise analysis could to be conducted if detailed information of original data is available. Second, a lack of original data of the reviewed studies limited our further evaluation of potential interactions, including the interactions between different genes and between gene and environment factors. Third, only English and Chinese documents and our unpublished study were included in this meta-analysis, while reports that were written in other languages and other unpublished data or ongoing studies were not available, which may cause certain publication bias in our meta-analysis. The last but not the least, the pooled sample size was relatively limited in this meta-analysis. Therefore, this meta-analysis could only preliminarily appraise the association of rs895819 polymorphism with currently-reported cancers. More studies are still required to get a more reliable result.

In summary, this meta-analysis indicated that the G allele of miR-27a rs895819 polymorphism may be associated with decreased risks of breast cancer, renal cell cancer and nasopharyngeal cancer, as well as a decreased cancer risk in Caucasians and AG heterozygote might be associated with an increased risk of digestive tract cancer. To confirm our findings, additional well-designed studies in diverse ethnic populations and functional studies regarding this SNP are required.

## Supporting Information

Table S1 **ORs (95% CI) of sensitivity analysis.**
(DOC)

Table S2 **Checklist of this meta-analysis.**
(DOC)
