# Supplementary material for: Pre-miR-27a rs895819A/G Polymorphisms in Cancer: A Meta-Analysis
Source: PLoS One. 2013 Jun 7;8(6):e65208. doi: 10.1371/journal.pone.0065208 (PMC3676439; doi:10.1371/journal.pone.0065208)
Supplement: Table S1 — ORs (95% CI) of sensitivity analysis. (DOC) [file pone.0065208.s001.doc]

| **Table S1**. **ORs (95% CI) of sensitivity analysis.** | | | | | |
| --- | --- | --- | --- | --- | --- |
| Excluding literature | AG *vs.* AA | GG *vs.* AA | Dominant model | Recessive model | A *vs.* G |
| one by one | OR (95% CI) | OR (95% CI) | OR (95% CI) | OR (95% CI) | OR (95% CI) |
| Over all | 1.009(0.877-1.163) | 0.895(0.791-1.013) | 0.975(0.864-1.101) | 0.911(0.809-1.026) | 0.936(0.887-0.987) |
| Qian Xu (2013) | 0.985(0.854-1.134) | 0.908(0.801-1.029) | 0.960(0.848-1.087) | 0.927(0.822-1.045) | 0.933(0.884-0.985) |
| Danni Shi (2012) | 1.046(0.906-1.207) | 0.903(0.793-1.028) | 1.003(0.886-1.135) | 0.908(0.802-1.027) | 0.950(0.989-1.004) |
| Renata Hezova (2012) | 1.015(0.874-1.178) | 0.892(0.786-1.012) | 0.977(0.860-1.111) | 0.908(0.804-1.025) | 0.934(0.885-0.986) |
| Irene Catucci (2012) | 1.030(0.875-1.187) | 0.908(0.791-1.043) | 0.991(0.862-1.138) | 0.918(0.805-1.048) | 0.943(0.889-1.000) |
| Yuan Zhou(2012) | 1.019(0.874-1.187） | 0.926(0.816-1.050) | 0.989(0.869-1.126) | 0.940(0.834-1.061) | 0.945(0.895-0.998) |
| Mingwu Zhang (2012) | 0.974(0.851-1.114) | 0.897(0.789-1.019) | 0.958(0.846-1.085) | 0.938(0.829-1.060) | 0.934(0.885-0.986) |
| Mingwu Zhang (2012) | 0.996(0.859-1.159) | 0.875(0.770-0.995) | 0.958(0.846-1.085) | 0.896(0.793-1.013) | 0.923(0.873-0.975) |
| Peirao Li (2011)† | 0.998(0.860-1.159) | 0.882(0.776-1.003) | 0.962(0.848-1.091) | 0.903(0.799-1.020) | 0.926(0.876-0.978) |
| Peirao Li (2011)※ | 1.040(0.892-1.213) | 0.914(0.801-1.043) | 0.999(0.877-1.138) | 0.919(0.811-1.043) | 0.952(0.899-1.010) |
| Ping Zhang (2011) | 1.000(0.863-1.160) | 0.887(0.782-1.006) | 0.964(0.850-1.094) | 0.906(0.803-1.021) | 0.930(0.881-0.982) |
| Wenzhang Wang (2011) | NA* | NA* | 0.987(0.867-1.124) | NA* | NA* |
| Qingmin Sun(2010) | 0.979(0.852-1.124) | 0.853(0.750-0.971) | 0.939(0.840-1.049) | 0.880(0.778-0.995) | 0.917(0.868-0.968) |
| Rongxi Yang(2010) | 1.043(0.898-1.212) | 0.898(0.780-1.034) | 1.000(0.878-1.140) | 0.886(0.775-1.014) | 0.952(0.897-1.010) |
| *NA: not applicable. † study for the liver cancer. ※ study for the nasopharyngeal cancer. | | | | | |
